# Trigeminal neuralgia with enlarged suprameatal tubercle: A case report

**DOI:** 10.4317/jced.61504

**Published:** 2024-05-01

**Authors:** Momoyo Kobayashi, Keita Takizawa, Andrew Young, Naoki Otani, Noboru Noma

**Affiliations:** 1 PhD student, Department of Oral Medicine, Nihon University school of Dentistry Tokyo, Japan; 2 MSD, DDS, Department of Diagnostic Sciences, Arthur Dugoni School of Dentistry, University of the Pacific, San Francisco, United States; 3 PhD, MD, Department of Neurological Surgery, Nihon University School of Medicine, Tokyo, Japan, 1-6 Kandasurugadai, Chiyoda-ku, Tokyo 101-8309, Japan; 4 PhD, DDS, Department of Oral Medicine, Nihon University school of Dentistry Tokyo, Japan

## Abstract

According to the International Classification of Orofacial Pain (ICOP), secondary trigeminal neuralgia can result from various conditions such as tumors in the cerebellopontine angle, arteriovenous malformation, and multiple sclerosis. This case report describes a 41-year-old woman with trigeminal neuralgia caused by narrowing of the cerebellopontine cistern due to an enlarged suprameatal tubercle. Carbamazepine treatment was initially effective, but became inadequate within a few months. Magnetic resonance imaging revealed compression of the trigeminal nerve by the superior cerebellar artery and an enlarged suprameatal tubercle. Microvascular decompression surgery was done to alleviate the neurovascular compression. Dentists should be aware of such anatomical factors contributing to trigeminal neuralgia, particularly in younger patients.

** Key words:**Trigeminal neuralgia, enlarged suprameatal tubercle, microvascular decompression.

## Introduction

According to the International Classification of Orofacial Pain (ICOP), recognized causes of secondary trigeminal neuralgia include tumors in the cerebellopontine angle (CPA), arteriovenous malformation, multiple sclerosis, skull-base bone deformity, connective tissue disease, arteriovenous malformation, dural arteriovenous fistula, and genetic causes of neuropathy or nerve hyperexcitability (1). Previously reported cases have described bony abnormalities such as petrous bone deformities or basilar impression, which resulted in compression of the trigeminal nerve or its nucleus (2).

Although the exact cause of neurovascular compression (NVC) remains unknown, certain anatomical variations, including vascular variations such as dolichoectasia, aberrant vessel loops, and tortuosity, have been associated with TN risk. Additionally, smaller cerebellopontine cistern cross-sectional areas and sharper trigeminal-pontine angles are linked to an increased risk of NVC. Here, we report a case of trigeminal neuralgia caused by the narrowing of the cerebellopontine cistern area due to enlargement of the suprameatal tubercle.

A 41-year-old woman presented with electric shock-like pain from the left side of her nose to her cheek. Approximately one month before visiting our orofacial pain clinic, the patient had visited a general dentist for such pain in the left anterior maxillary gingiva. The paroxysms occurred 3 to 4 times a day, with triggers being talking, meals, facial washing, and tooth brushing. The duration of the pain ranged from 1 to 10 seconds. A neurological examination at Nihon University Hospital revealed stabbing pain lasting approximately 10 seconds, induced by light touch to the left side of the face. No additional abnormalities were found.

She was diagnosed with trigeminal neuralgia and started on carbamazepine 100 mg twice daily, which successfully relieved the paroxysmal pain. However, several months later, pain control lessened, and the dosage was increased to 300 mg three times a day. Despite this adjustment, the medication was no longer effective, leading to a referral to the neurosurgery department.

## Case Report

-MRI findings

**Figure 1:**
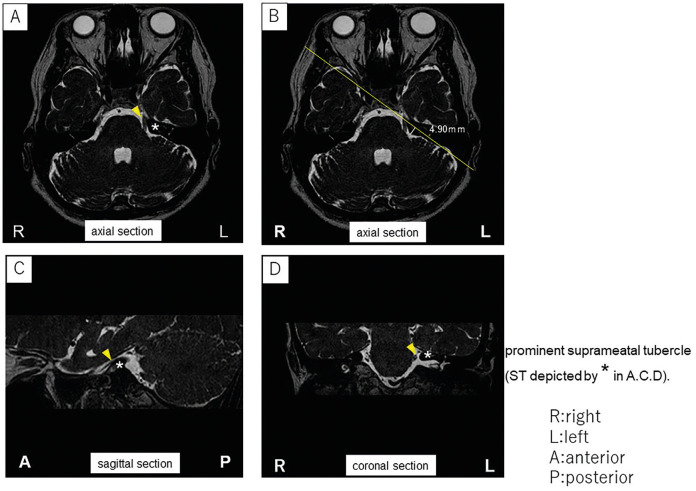
In the cerebellopontine angle area, a prominent suprameatal tubercle (ST) was observed, depicted by * in T1-weighted, contrast-enhanced axial (A), sagittal (C), and coronal (D) views, with contact noted between the superior cerebellar artery and the trigeminal nerve root (yellow arrows). B: The height of ST on the MRI was 4.90 mm.

Magnetic resonance imaging (MRI) revealed that the superior cerebellar artery (SCA) was causing neurovascular compression at the trigeminal nerve root entry zone. Furthermore, an enlarged suprameatal tubercle was noted on the left side, compressing the neurovascular complex of the trigeminal nerve (Fig. 1A-D).

-Operation

**Figure 2:**
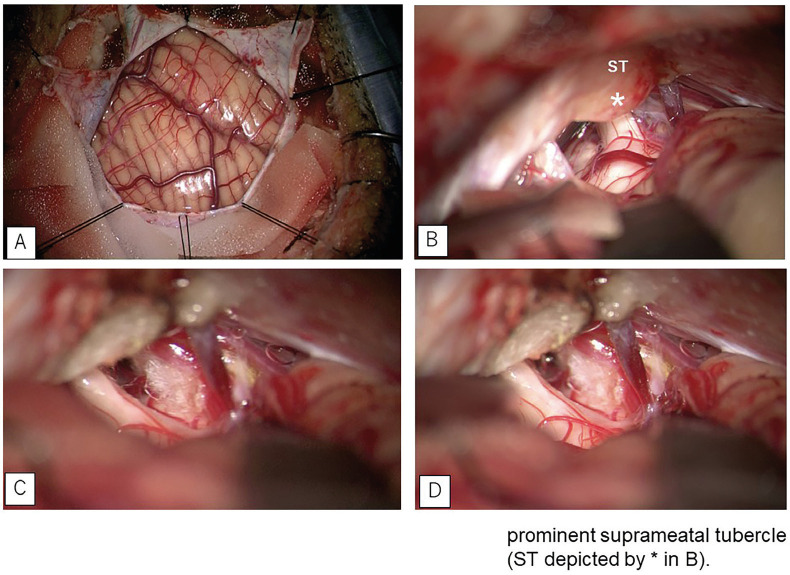
A: The dura mater was incised linearly at the base of both the transverse sinus and sigmoid sinus, with an additional tapered incision extended towards the transverse-sigmoid junction. B: Intraoperative microscopic view showing prominent the suprameatal tubercle (ST) covering the cisternal segment of trigeminal nerve. C,D: The entire length of the trigeminal nerve can be seen after drilling of the ST. Note the pressure mark over the nerve caused by ST compression.

Following the patient’s informed consent, left-sided microvascular decompression (MVD) was performed. A retrosigmoid craniotomy allowed dissection to expose the structures at the CPA. Initially, the IV and VIII were clearly visible, but only the proximal portion of the V nerve was evident within the surgical field (Fig. 2A,B). The site of neurovascular conflict, where the SCA traversed the V nerve, was completely obscured by a prominent suprameatal tubercle. To access the site of neurovascular conflict, the suprameatal tubercle was carefully drilled using a diamond drill.

-Partial removal of the suprameatal tubercle allowed further visualization of the surrounding arachnoid adhesions, and revealed dorsal compression of the trigeminal nerve root entry zone by the SCA (Fig. 2.C,D). Following dissection of the arachnoid adhesions, the SCA was detached from the trigeminal nerve root, concluding the MVD.

## Discussion

TN is widely recognized as the predominant craniofacial neuropathic pain. It is considered one of the most severe forms of pain a person can endure, with an estimated incidence ranging from 4 to 13 individuals per 100,000 annually (3).

Trigeminal neuralgia onset typically occurs in individuals aged 50 or older, with an average onset age of 51.3 years for males and 52.9 years for females (4).

In this case report, we present a less-common occurrence of trigeminal neuralgia onset at the age of 41 in a female patient. TN in younger women may arise due to constriction of the CPA cistern, forcing the intracranial vessels to meander and bend, leading to neuralgic manifestations. A diminished trigeminal pontine angle could influence the development of NVC along the medial aspect of the trigeminal nerve. This is because a reduction in the angle leads to a decrease in the cisternal space between the trigeminal nerve and the surface of the pons, which is a crucial area where NVC is thought to occur (5). A smaller cisternal space may bring the nerve and blood vessels into closer proximity, potentially increasing the incidence of NVC. These findings indirectly support the idea that a smaller trigeminal pontine angle might heighten the risk of NVC on the medial side of the trigeminal nerve, and thereby increase the risk for TN. Therefore, a reduced trigeminal pontine angle could be considered a predisposing factor for NVC in TN patients (5).

In 1970, Obrador *et al*. reported a rare case of right-sided TN in a 38-year-old woman due to petrous bone asymmetry and unilateral basilar impression (6). Relief was achieved through a partial rhizotomy using a subtemporal approach. These anatomical factors have also been noted in several studies on patients with TN. Erbay *et al*. reported that in their study of 36 TN patients, the nerve diameters and cross-sectional areas were 20% and 28% smaller, respectively, on the symptomatic side compared to the asymptomatic side (7). Similarly, Rasche *et al*. also suggested that a smaller cross-sectional area might be linked to a higher incidence of NVC between the trigeminal nerve and surrounding vessels (8). In this case, the suprameatal tubercle protruded on the symptomatic side, resulting in a noticeably smaller volume of the CPA compared to the asymptomatic side, which is consistent with previous reports.

Petrous endostosis, or a prominent suprameatal tubercle, has an estimated incidence rate of approximately 5% (9). The limited available literature suggests significant variability in the morphology of the petrous bone in this area. In a study investigating the microsurgical anatomy of the suprameatal tubercle using 15 cadaveric heads (30 cochlear implantation petrous apices), Seoane and Rhoton found that the average height of this bony prominence was approximately 4.1 mm (ranging from 2.8 to 6.0 mm) above the posterior surface of the petrous ridge (10). Conversely, Oiwa *et al*. reported mean values ranging from 1.4 to 1.7 mm based on the analysis of 106 patients who underwent 3D CT scans of the skull, with only 5.2% showing heights greater than 3 mm (9). In our presented patient, we observed a height of 4.90 mm in the preoperative MRI (Fig. 1B).

From a neurosurgical perspective, the prominent bony protrusion encountered during the retrosigmoid approach, known as the suprameatal tubercle, is situated above the superior margin of the internal auditory meatus (10). Drilling into the suprameatal tubercle provides access to Meckel’s cave and the posterior aspect of the middle cranial fossa. However, in cases where the offending vessel underlying the prominent suprameatal tubercle is not visible, or when the entire length of the trigeminal nerve cannot be visualized, it may become necessary to drill into this structure during microvascular decompression of the trigeminal nerve, especially when it is heavily calcified and covered by an enlarged tubercle (10). During our surgical exploration, we encountered a bony tumor extending from the posterior wall of the internal auditory canal to the suprameatal tubercle, obstructing visualization of the entire length of the trigeminal nerve. After performing bone drilling, we identified the peripheral branch of the SCA in contact with the trigeminal nerve. Further bone drilling revealed adhesion of the central portion of the SCA to the trigeminal nerve itself, causing displacement of the trigeminal nerve axis.

In conclusion, in cases of younger patients with trigeminal neuralgia, it is important to consider secondary trigeminal neuralgia due to tumors in the CPA, arteriovenous malformation, and multiple sclerosis, based on the clinical findings. The utility of an MRI for trigeminal neuralgia lies firstly in identifying the responsible vessels, and secondly in excluding secondary trigeminal neuralgia. Additionally, dentists should be familiar with the anatomy around the posterior cranial fossa, as well as the potential for trigeminal neuralgia to arise from CPA compression due to a prominent suprameatal tubercle, as observed in this case.

## Data Availability

The datasets used and/or analyzed during the current study are available from the corresponding author.
